# Quantitative immunohistochemistry analysis of breast Ki67 based on artificial intelligence

**DOI:** 10.1515/biol-2022-1013

**Published:** 2024-12-31

**Authors:** Wenhui Wang, Yitang Gong, Bingxian Chen, Hualei Guo, Qiang Wang, Jing Li, Cheng Jin, Kun Gui, Hao Chen

**Affiliations:** Department of Pathology, Hangzhou Women’s Hospital, Hangzhou, 310008, Zhejiang, China; Ningbo Konfoong Bioinformation Tech Co., Ltd, Ningbo, China; Department of Pathology, Hangzhou Women’s Hospital, 369 Kunpeng Road, Shangcheng District, Hangzhou, 310008, Zhejiang, China

**Keywords:** artificial intelligence, breast cancer, algorithms, deep learning, image detection, Ki-67

## Abstract

Breast cancer is a common malignant tumor of women. Ki67 is an important biomarker of cell proliferation. With the quantitative analysis, it is an important indicator of malignancy for breast cancer diagnosis. However, it is difficult to accurately and quantitatively evaluate the count of positive nucleus during the diagnosis process of pathologists, and the process is time-consuming and labor-intensive. In this work, we employed a quantitative analysis method of Ki67 in breast cancer based on deep learning approach. For the diagnosis of breast cancer, according to breast cancer diagnosis guideline, we first identified the tumor region of Ki67 pathological image, neglecting the non-tumor region in the image. Then, we detect the nucleus in the tumor region to determine the nucleus location information. After that, we classify the detected nucleuses as positive and negative according to the expression level of Ki67. According to the results of quantitative analysis, the proportion of positive cells is counted. Combining the above process, we design a breast Ki67 quantitative analysis pipeline. The Ki67 quantitative analysis system was assessed on the validation set. The Dice coefficient of the tumor region segmentation model was 0.848, the Average Precision index of the nucleus detection model was 0.817, and the accuracy of the nucleus classification model was 96.66%. Besides, in clinical independent sample experiment, the results show that the proposed breast Ki67 quantitative analysis system achieve excellent correlation with the diagnosis efficiency of doctors improved more than ten times and the overall consistency of diagnosis is intra-group correlation coefficient: 0.964. The research indicates that our quantitative analysis method of Ki67 in breast cancer has high clinical application value.

## 1 Introduction

Breast invasive ductal carcinoma (IDC) is one of the common malignant tumors with high mortality in women [1,2,3]. The antigen (Ki67) recognized by monoclonal antibody Ki67 is one of the common clinical diagnostic biomarkers of breast cancer pathology. Ki67 is a cell cycle-related nuclear protein that has no tissue specificity and is only expressed in proliferating cells, which can be used as a basis for accurately predicting the proliferation status of tumor cells and its malignancy. Quantitative assessment of tumor biomarkers serves as a main direction of precise pathology diagnosis. The precision medicine of breast cancer mainly includes precise diagnosis and precise medicine, of which the diagnosis of breast cancer Ki67 is a crucial process. The indicators of Ki67 in breast cancer are closely related to the clinic pathological characteristics and molecular subtyping, which is not only a reference indicator for the prognosis and individualized treatment of breast cancer patients [4,5] but also of great significance in distinguishing the subtypes of intraluminal breast cancer and determining the optimal treatment scheme [6,7]. It has important clinical significance in guiding pathology diagnosis, pathology classification, guiding clinical medication and treatment, and judging prognosis and therapeutic efficacy. It is an important step in the process of tumor diagnosis and treatment. However, the Ki67 quantitative score of breast cancer is difficult to interpret, and the interpretation consistency between laboratories and pathologists is generally poor. The quantification of eye inspection is highly subjective, highly variable, poor repeatability, and inefficient. It lacks a strict and unified understanding system and standard. Besides, it is difficult and time-consuming for pathologists to diagnose the quantitative markers of a single cell under the microscope. In response to these problems, the consensus on the Ki67 assessment 2021 edition (the International Breast Cancer Ki67 Working Group) clearly pointed out that Artificial Intelligence (AI) assistive and automation scoring may be a feasible solution to the main points of Ki67 assessment [8].

In clinic pathological practice, the factors causing poor repeatability of interpretation on Ki67 immunohistochemistry (IHC) results in breast cancer mainly include interpretation mode, interpretation region, and the selection of interpretation cells. The method used by pathologists in the diagnosis of Ki67 is usually sampling estimation rather than comprehensive assessment, which is highly subjective, resulting in the deviation between the counting results and its real value. Second, there is no strict and unified standard for the counting method of Ki67 in clinic pathological practice, while some diagnostic details are still controversial. The 2011 Breast Cancer Ki67 International Working Group Recommended Assessment Guideline suggested that 1,000 invasive cancer cells in a representative region should be selected and counted after the assessment of the whole slide, not less than 500 invasive cancer cells [9]. However, in practical work, counting more than 500 cancer cells per slide will take a lot of time and the controllability is poor, if not infeasible. In addition, some pathologists are used to making gross estimates at low magnification, while others prefer to make careful identification at high magnification. For slides with a relatively uniform distribution of Ki67, the mean value of any selected field is estimated, and the difference in the evaluation index between pathologists is relatively small. However, for slides with large Ki67 heterogeneity, some doctors still estimate the Ki67 index based on the average of several views while some assess the Ki67 index based on hot spots and the identification and selection of regions for hot spots varies between pathologists. No matter which method is adopted, most pathologists would often not use 40× magnification for observation and counting throughout the diagnosis. In addition, after manually counting the Ki67 positive cells in several fields of view, some pathologists are used to calculating the final result based on average value, while others believe that the highest of these values should be reported. Besides, different pathologists will have different counts for the same selected region and the consistency of repetitive counts for the same region in different time periods is poor even for the same doctors. The final results are affected by subjective factors such as the time period of the doctor’s work and the degree of visual fatigue at work. These counting habits and methods that have not yet been unified are an important reason why the final results of manual Ki67 counting may be biased within a certain range.

As a pathology diagnosis work based on the empirical and cumulative disciplines, the identification of positive tumor cell counting in IHC slides is affected by objective and subjective factors such as the pathologist’s qualifications, work experience, and the accumulation of slide viewings in the clinical practice of daily Ki67 diagnosis. In the interpretation of Ki67 positive tumor cells, the pathologists with low seniority, less accumulated number of slide viewings, and work experience have a great difference from those with high seniority and rich experience in the interpretation of slides with more interference. The Ki67 gold standard is derived from an evaluation of the percentage of tumor cells counted for Ki67 in whole IHC slide. AI approaches performing cell counts not only meet the International Breast Cancer Working Group standards but also goes beyond the range of cell counts recommended by the Working Group. Research results [10] showed that the AI-based evaluation method for Ki67 was superior to manual evaluation in both sensitivity and specificity, while only a few regions can be selected to make evaluation in clinical pathological practice. Therefore, AI methods counting for Ki67 in breast cancer are more likely to standardize the interpretation region (multi-region average), which is very important for the clinical application of Ki67 [11]. A similar study of manual vs AI in counting of Ki67 reported a clear advantage in correlation and consistency of AI evaluation [12]. AI software based on whole slide imaging (WSI) is a potentially effective method for Ki67 evaluation, with higher consistency and accuracy than visual assessment [13,14].

With the continuous development of computer technology, the combined application of digital pathology and artificial intelligence has been proven to exhibit high accuracy and repeatability in the evaluation of Ki67 [15]. For instance, Joseph et al. [16] developed a Proliferative Tumor Marker Network (PTM-NET), using convolutional neural networks to objectively annotate tumor areas marked with Ki67 in digital pathology images of breast cancer. However, the issue of overlapping cells between adjacent cells was not sufficiently considered. Negahbani et al. [17] created the SHIDC-BC-Ki67 dataset, utilizing the PathoNet semantic segmentation network for Ki67 detection and employing a post-processing solution to mitigate interference caused by overlapping cells. Valkonen et al. [18] employed dual staining of fluorescently labeled cytokeratin and Ki67, along with sequentially stained hematoxylin and eosin (HE) images for training, fine-tuning a VGG network for semantic segmentation. This method also overlooks the relationships between neighboring cells. Niyas et al. [19] proposed an improved LadderNet architecture for segmenting immune-positive elements from ER, PR, HER2, and Ki67 biomarker slides. The approach involves an ensemble of multiple fully convolutional neural networks, with each sub-network responsible for the segmentation of one biomarker. Feng et al. [20] used image registration to align immunohistochemistry images with their corresponding HE images, classifying tiles/patches of WSI to ultimately assemble the entire WSI for Ki67 scoring. However, this method carries the risk of missing global information and failing to capture overall cell information. In addition, Priego-Torres et al. [21] utilized a deep learning-based instance segmentation architecture for automatic quantification of nuclear and membrane biomarkers applied to IHC-stained slides. However, relying solely on a single instance segmentation network makes it challenging to distinguish between nuclear and cytoplasmic markers. Furthermore, Catteau et al. [22] and Robertson et al. [23] employed the QuPath [24] software for Ki67 measurement. QuPath software remains sensitive to hyperparameters in terms of cell detection, cell segmentation, and protein marker determination, requiring adjustments by pathology experts, thus categorizing it as semi-automatic measurement software with relatively lower accuracy.

In summary, current artificial intelligence methods still have some shortcomings in the assessment of Ki67, including insufficient utilization of relationships between adjacent cells, cell positional information, global information of WSI, and morphological features of cell nuclei and cytoplasm. Additionally, the limited expression capability of networks, sensitivity to hyperparameters, and the need for expert intervention still render these methods semi-automatic. Further research and improvement are needed to enhance the accuracy and repeatability of Ki67 assessment. An AI-based evaluation method of Ki67 was developed [25], and the results showed that the method can assist pathologists to achieve higher consistency (intra-group correlation coefficient, ICC = 0.942 [95% CI: 0.926, 0.957]).

In order to further make better use of AI-assisted diagnosis in the field of pathology, it is critical to develop and improve an automated and efficient method to identify and diagnose positive and negative nucleus in Ki67 image. We have designed an efficient system based on deep learning algorithm framework for the further study on the automatic quantitative method of breast Ki67 IHC pathology slide image.

## 2 Materials and methods

### 2.1 Data collection and annotation

**Figure 1:**
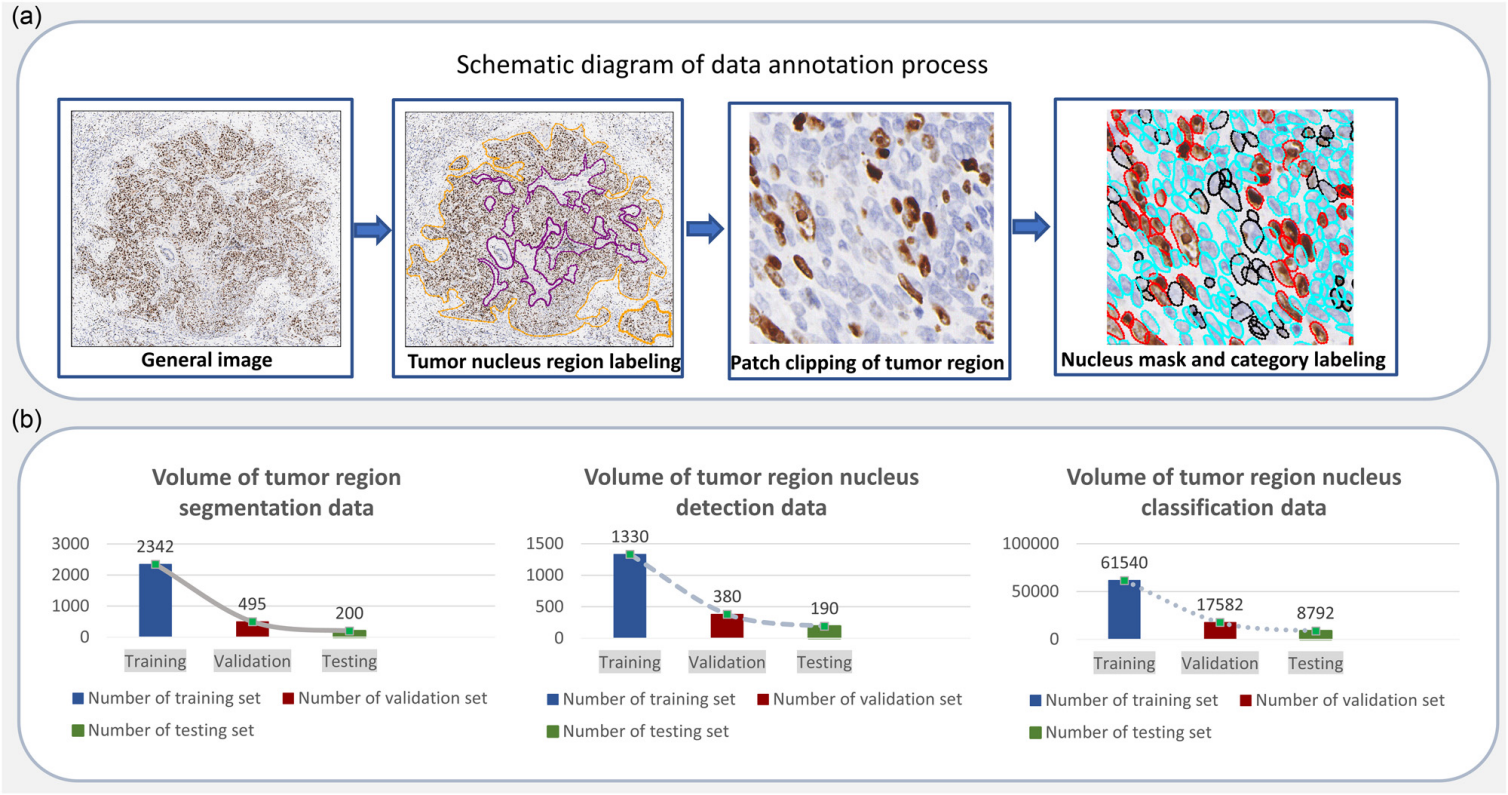
Dataset generation and distribution map of breast Ki67 analysis system. (a) The flow chart of the dataset label annotation: first annotating the tumor region mask (the delineation of the tumor area is indicated by an orange curve, whereas the purple area signifies the normal tissue region), then extracting the mask region patch image and annotating the cell region mask and cell category (the red curve precisely outlines positive cells, the blue curve outlines negative cells, and the black curve designates the boundaries of cells that are indistinct in nature) and (b) the data volume distribution of each model training, validation, testing (in order as follows: tumor region segmentation model data volume distribution, cell detection model data volume distribution, and cell classification model data volume distribution).

The data source for this study was the Hangzhou Obstetrics and Gynecology Hospital. A total of 400 breast Ki67 WSI pathology slides were obtained through scanning by KFBIO Scanner. The WSI pathology slides were divided into 8:2, with 320 slides used for model data annotation and 80 slides used for model inference. The data division is based on patients, meaning that the inference dataset used for testing is entirely independent of the annotation dataset. There are no common patients, and any WSI region comes from the annotation dataset used for training. The annotated dataset consists of two levels, which are the tile level and the WSI level. At the whole slide image level, we have explicitly annotated the specific values of ki67 positivity and drawn the curve graph of the tumor area. At the tile level, we have carefully annotated the contours of the cells and further specified the classification attributes of the cells, i.e., whether the cells belong to positive, negative, or uncertain categories. In contrast, the dataset used for inference only contains Ki67 percentage annotations. For the WSI to be annotated, pre-processed slicing was used to cut the slide into 1,536 × 1,536 pixel images. Then, the dataset for the tumor region segmentation model, tumor region nucleus detection model, and tumor region nucleus classification model were constructed, respectively. The dataset was divided in the ratio 7:2:1 (training:validation:test), while the training set was used for model training, the validation set was used to evaluate the effect of the model, and the test set was used for model inference. Specifically, the division of the annotated dataset is mixed based on tiles/patches. This implies that the training set, validation set, and test set in this portion may come from the same patient, but they cannot originate from the same WSI region. The data annotation quantities of each model involved in the AI-based breast Ki67 analysis system are shown in Figure 1b.

Following the specific process of data annotation shown in Figure 1a, the labeling team, which includes labeling physicians, pathologists, authoritative experts, and technicians, is well-versed in the operation of the labeling software. They are responsible for establishing labeling rules and ensuring a unified understanding of these rules. The physicians involved in annotation possess the qualifications to perform film reading tasks in the pathology department of a tertiary first-class hospital for over 5 years and are capable of issuing pathological diagnosis reports independently. In addition, a senior expert in this professional field is required to review, check, and correct missing or wrong labels in a timely manner. The data required for the tumor region segmentation framework mainly label the tumor region, while the data required for the nucleus detection framework in the tumor region mainly label the instance information of the nucleus in the labeled tumor region and the data required for the nucleus classification framework in the tumor region mainly label the category of the nucleus. In particular, due to the large amount of data required for the training of deep learning models, a semi-automatic annotation method was used to improve the efficiency of data calibration. Based on the existing tumor region segmentation model, nucleus detection model, and nucleus classification model, inference was performed on the unannotated data. The information on the inference results was obtained and transferred to the annotation format required by annotation software as the pre-annotation results. Then, the pre-annotation results were manually revised to modify the incorrect pre-annotation results. Finally, the experts reviewed the annotation results to ensure the quality of the annotation.


**Informed consent:** Informed consent has been obtained from all individuals included in this study.
**Ethical approval:** The research related to human use has been complied with all the relevant national regulations, institutional policies and in accordance with the tenets of the Helsinki Declaration, and has been approved by the Ethical Review Committee of Hangzhou Women’s Hospital ([2021]A-8-03).

### 2.2 Design of technical framework

**Figure 2:**
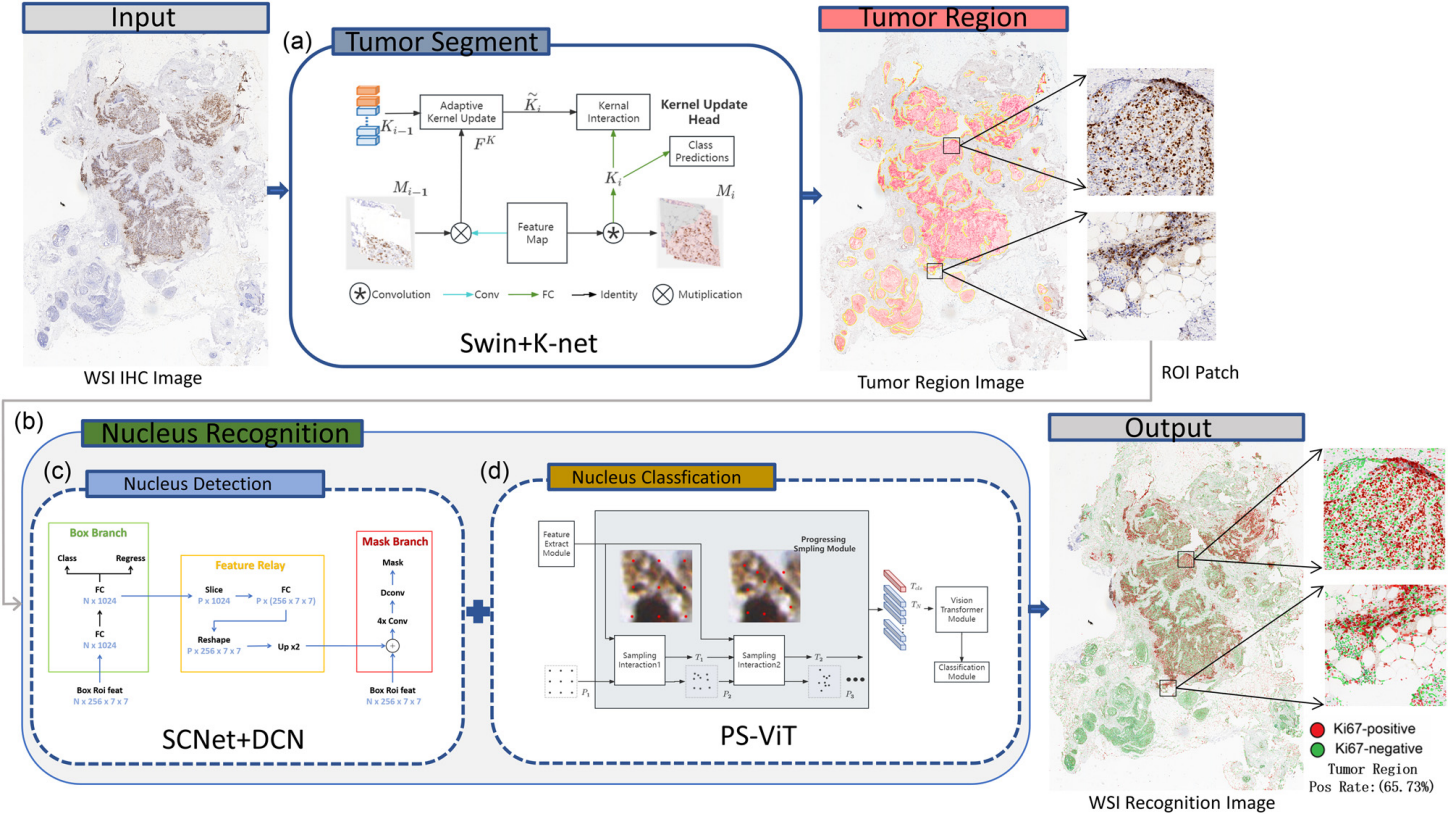
Overall framework of the WSI quantitative analysis system for breast Ki67 pathology image. ROI refers to the region of interest. This term is used to describe the parts of pathological images that are important for diagnosis and analysis. ROI patch refers to a small area segmented from the WSI image that contains important diagnosis information. Swin+K-Net, SCNet+DCN, and PS-ViT are all deep learning methods for specific tasks. (a) Tumor segment. (b) Nucleus recognition. (c) Nucleus detection. (d) Nucleus classification.

For the quantitative analysis of Ki67 WSIs of breast cancer, we designed a technical pipeline based on AI technology to build a WSI tumor region rapid quantitative analysis system for breast Ki67 IHC images. The system framework is shown in Figure 2.

The assessment of Ki67 positive cells should be with the tumor regions. Our WSI tumor region rapid quantitative analysis system adopts deep learning image segmentation algorithm Swin+K-Net [26], for tumor region segmentation to automatically and rapidly identify the region where the tumor cells are located in the breast Ki67 pathology image. Then, the deep learning instance segmentation algorithm SCNet+DCN+LSJ is applied in this system to detect cells in the tumor region, obtaining the minimum bounding box (MBB) information of the cell location and cell mask region. Thus, it uses the deep learning target classification algorithm PS-ViT to classify the cells in the MBB to identify whether the cell is positive or negative. Finally, cell identification in tumor regions of the breast Ki67 pathology image is achieved and quantitative analysis is also performed to calculate the percentage of positive cells. Above all, the final result of the WSI of the breast Ki67 pathology image is determined.

### 2.3 Tumor region segmentation

Tumor region segmentation is a fundamental step of the quantitative breast Ki67 analysis system, which can segment the tumor regions, eliminating non-tumor regions and blank regions, thereby improving the rate of tumor cell identification.

**Figure 3:**
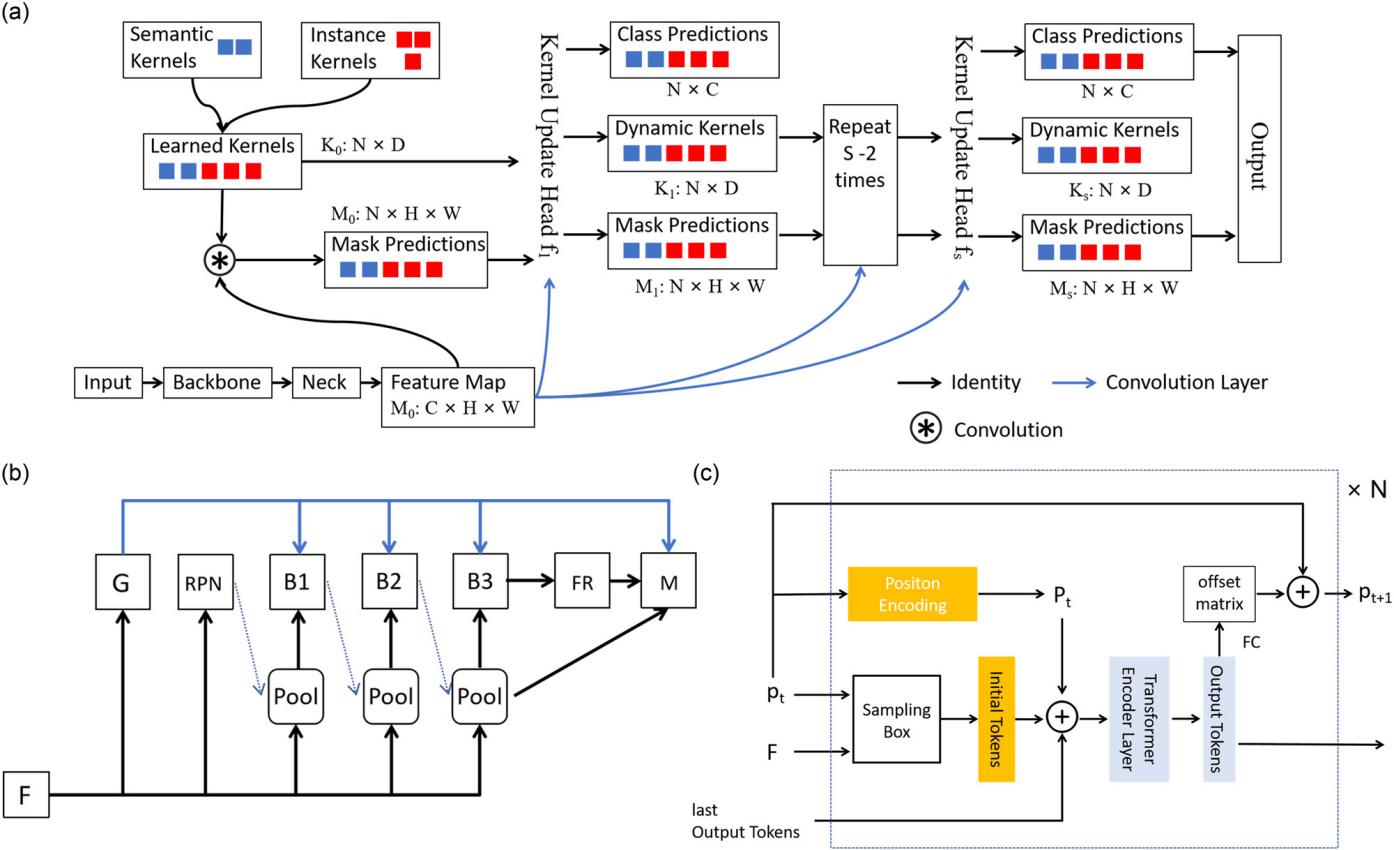
Framework diagram for each model of the breast ki67 analysis system. (a) The framework diagram of the K-Net algorithm for tumor region segmentation. (b) The framework diagram of SCNet algorithm for tumor region nucleus detection. (c) The framework diagram of PS-ViT for tumor region nucleus classification.

We used a semantic segmentation method combining Swin and K-Net [26] for tumor region segmentation, considering the K-Net network achieved state of the art performance in the field of image segmentation. Different from FCN [27], PSPNet [28], and DeepLab [29,30], K-Net is comprised of a set of kernels responsible for generating semantic masks. Each kernel is tasked with a specific semantic category, as illustrated in the network framework shown in Figure 3a. For more details on the K-Net network structure, please refer to the “Tumor Region Segmentation” section in the supplementary material.

K-Net can adapt to different frameworks by making the semantic convolution kernel dynamic. We combined K-Net with Swin Transformer to apply tumor region segmentation on breast ki67 IHC pathology image and achieved excellent results, which well separated the main breast tumor regions. At present, in terms of diagnosis, the quantitative analysis of breast Ki67 pays more attention to the proportion of positive nucleus in the tumor region. Therefore, we first take the tumor region segmentation to extract the tumor region, and filter out the detection of nucleuses in the non-tumor region, which can not only significantly improve the efficiency of nucleus recognition in the WSI but also the accuracy of diagnosis can be improved. The way we adopt is much closer to the diagnosis of physician.

### 2.4 Nucleus detection in tumor region

Nucleus detection is another critical step of the quantitative breast Ki67 analysis system, which is performed on a segmented tumor region and focuses on detecting nucleuses within the tumor region. It is a prerequisite for the quantitative analysis of breast Ki67 to be achieved.

For the nucleus detection in breast Ki67 pathology image, we adopt a SCNet network combined with a variable convolutional DCN network and large-scale jitter (LSJ) data enhancement. The current cascade architecture has brought significant performance improvements in the instance segmentation, e.g., Cascade R-CNN [31], Mask R-CNN [32], and Hybrid Task Cascade [33] (HTC). However, the problem of the limitations of existing cascade methods for instance segmentation with mismatched distributions of training and inference samples has always affected the effectiveness of instance segmentation. SCNet [34] introduces a sample consistency network, as shown in Figure 3b to ensure that the training sample intersection over union (IoU) distribution is close to the IoU distribution at inference. The network combines feature relaying and overall contextual information to further enhance the interrelationship between the sub-tasks of classification, detection, and segmentation. In this way, the problem of the limitations of existing cascade methods, for instance, segmentation with mismatched distributions of training and inference samples is addressed. Compared to the powerful Cascade Mask R-CNN instance segmentation network, the accuracy of the SCNet network has been improved, while the speed has also been significantly improved. For more details on the SCNet network structure, please refer to the “Nucleus Detection in Tumor Region” section in the supplementary material.

We apply SCNet network combined with variable convolutional DCN network and multi-scale training and large-scale dithering data enhancement to detect nucleus in the tumor region of breast Ki67 IHC pathology image. Through training nucleus instance segmentation model, the nucleus MBB location information and nucleus segmentation mask information in the tumor region can be obtained. The location of nucleus in the tumor region of the breast Ki67 IHC pathology images has been well detected, providing an accurate reference for the quantitative analysis of Ki67 IHC pathology image diagnosis.

### 2.5 Nucleus classification in tumor region

Nucleus classification is the final essential step in our study. This is mainly used to distinguish the detected nucleuses categories and serves an important component of determining the proportion of positive nucleus in breast Ki67 quantitative analysis. For the classification model to differentiate positive and negative nucleus of breast Ki67 pathology image, we apply the PS-ViT [35] network which has achieved good performance. Transformer, a network with powerful global coding capabilities, has recently been widely applied to computer vision tasks. For example, ViT [36] used the transformer directly to solve an image classification task. To process two-dimension image data, ViT simply segmented the image and mapped it into a one-dimension sequence. This simple segmentation leaves the inherent structural information of the image lost, making it difficult for the network to focus on important object regions. To address this problem, PS-ViT proposes an iterative progressive sampling strategy to locate important regions, as shown in Figure 3c, where the sampling locations are updated at each iteration using global information. Therefore, it allows the network to gradually focus on the information of interest. The specific structure of the PS-ViT network can be found in the “Nucleus Classification in Tumor Region” section in the supplementary material.

**Figure 4:**
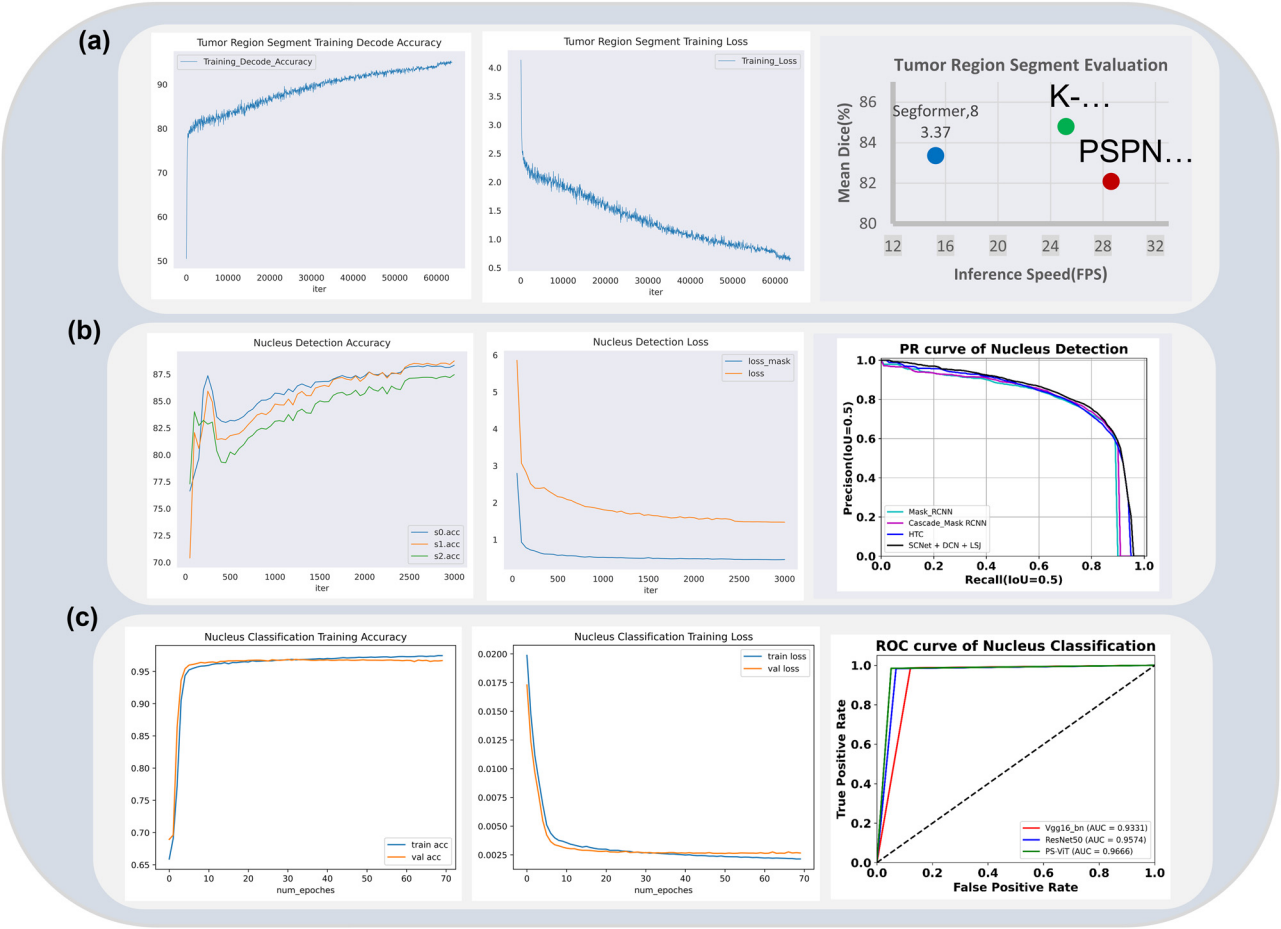
Comparison of the assessment metrics of each model of the breast Ki67 analysis system. (a) The assessment of the tumor region segmentation model. (b) The assessment of the tumor region nucleus detection model. (c) The assessment of the tumor region nucleus classification model.

We extracted the nucleus from the detected nucleus MBB in the tumor region, using the labeled category of the nucleus as ground truth. The distribution of the dataset is shown in Figure 4c. By using the PS-ViT network, we train a classification model, which is able to automatically distinguish whether the nucleuses detected in the tumor region of breast Ki67 pathology images are negative or positive. Thereby, it completed the accurate identification of positive and negative nucleus.

### 2.6 Training implementation

The algorithm framework involved in the Ki67 breast quantitative analysis system is achieved based on Python 3.7 and Pytorch 1.8.0. For all training samples, data augmentations such as flips and rotations are used to increase data diversity. We train our models on a NVIDIA RTX 3090 *4 GPU with 24GB memory. The input image size of the tumor region segmentation and tumor region nucleus detection are set as 512 × 512, while the input image size of tumor region nucleus classification is 48 × 48. For more training details, please refer to the “Training Implementation” section in the supplementary material.

### 2.7 Full image inference and prediction

AI-based studies of Ki67 breast pathology images are mainly carried out on WSI, whose resolution scale are often very large, often reaching the 100,000 × 100,000 level, making it difficult for machines to achieve direct inference. We used a sliding window to crop the whole image into patches for WSI inference. Then, the patch results were stitched and mapped to the full image to achieve WSI inference.

Specific process is as follows: First, set a window sliding block with the size of 1,536 × 1,536 and the stride of 300 for the WSI to obtain the patches. Then, applied the deep learning tumor region segmentation model to obtain the tumor region in the patch image. Next detected the nucleus by using the deep learning instance segmentation model to get the location information MBB and the nucleus mask in the tumor region. After that, classified the nucleus in MBB through the nucleus classification model and identified the nucleus categories. Finally, the identification results of nucleuses in the tumor region of patch were spliced and mapped to the original image. During the splicing process, non maximum suppression was used to suppress the repeated prediction of the target in the overlapping region. Based on the above steps, the nucleus identification results of the WSI were obtained. The number of positive nucleus and negative nucleus in the tumor region of the WSI, as well as the proportion of positive nucleus, was counted. Ultimately, the AI-based diagnosis results of the breast Ki67 pathology images were completed.

### 2.8 Evaluation metrics

Our breast Ki67 quantitative analysis system includes three deep learning frameworks in different fields. In order to evaluate the effect of each deep learning model, we respectively evaluated it on the corresponding model validation set. Each model adopts the corresponding evaluation method. Besides, to verify the clinical effect of our breast Ki67 quantitative analysis system, we evaluate the effect on the clinical data and compared with the results of the physicians. The relevant evaluation methods are described as follows.

**Figure 5:**
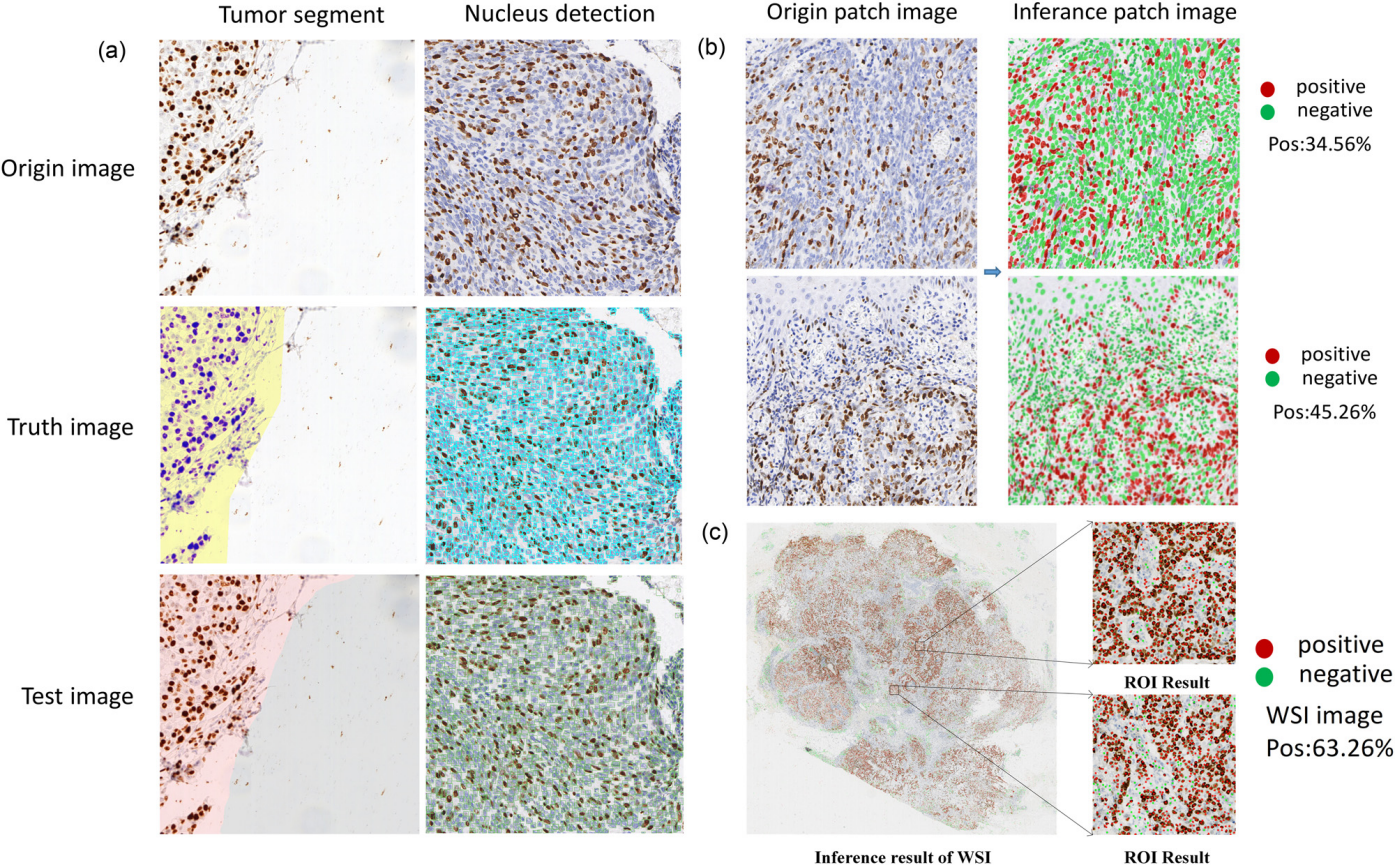
Visualized inference results of breast Ki67 analysis system. (a) The visualization of the inference results of tumor segment and nucleus detection in patch. (b) The visualization of the inference of nucleus recognition in patch. (c) The visualization of the WSI inference of the breast Ki67 case.

(a) The evaluation method of the tumor region segmentation model was based on mDice (an ensemble similarity measure function), which is the Dice coefficient that is usually used to calculate the similarity of two samples (with values ranging from [0, 1]).
(1) \[\text{Dice}=\frac{2| X\cap Y| }{| X| +| Y| },]\]
  where *X* and *Y* represent the set of predicted labels and true labels, respectively. |*X*∩*Y*| is the true intersection of the labeled pixel and the predicted pixel, |*X*| and |*Y*| are the number of true values in the predicted pixel and the labeled pixel, respectively. The calculation of mDice is to calculate Dice of all categories, and then take the average value.
(b) The nucleus instance segmentation model was evaluated by adopting the mAP index, which is commonly used in the field of instance segmentation. To calculate mAP, first the instance segmentation Precision and Recall need to be calculated. Precision is the proportion of samples with positive predictions that are actually positive, which can be thought of as the ability of the model to find the correct data. Recall refers to the proportion of the samples predicted positive while it is actually positive in the total positive samples. Recall can be regarded as the ability of the model to detect target data in the dataset. The calculation formula is as follows:
(2) \[\text{Precision}=\frac{\text{TP}}{\text{TP}+\text{FP}},]\]
(3) \[\text{Recall}=\frac{\text{TP}}{\text{TP}+\text{FN}}.]\]
  TP (True Positive) means the prediction is positive and the truth is positive, and the prediction is correct. FP (False Positive) means the prediction is negative and the truth is positive, and the prediction is wrong. FN (False Negative) means the prediction is negative and the truth is positive, and the prediction is wrong.
  Second, a concept in the field of object detection need to be illustrated, IoU, which is the ratio of intersection and merge, indicating the degree of overlap between the prediction box and the original annotation box. The ideal situation is the complete overlap, where the ratio is 1. IoU is used to measure the accuracy of the prediction box. In general, the ratio of IoU above 0.5 is considered as a good result.
  Next we draw the Precision and Recall curve, which is the P–R curve. The Precision and Recall of each predict result are calculated and the curve is drawn according to the relationship. As shown in Figure 5.
  Finally, on the basis of the Precision–Recall curve, a numerical metric can be obtained by calculating the average of the Precision values corresponding to each Recall value, used to measure how well the trained model is able to detect the category of interest. The calculation formula is as follows:
(4) \[\text{AP}=\mathop{\sum }\limits_{i=1}^{n-1}({r}_{i+1}-{r}_{i}){P}_{\text{interp}}({r}_{i+1}),]\]
  where *n* represents the number of samples. *r*_1_, *r*_2_, …, *r*_*n*_ are the recall values corresponding to the first interpolation of the precision interpolation segment in ascending order. *P*_interp_(*r*_*i*+1_) is the maximum value of the sample after taking the *r*_*i*+1_th point.
  AP value is calculated for only one category. Once the AP is obtained, mAP is calculated for all categories and then averaged. mAP measures how well the trained model is at detecting all categories. Calculation formula is as follows:
(5) \[m\text{AP}=\frac{{\sum }_{i=1}^{k}{\text{AP}}_{i}}{K}.]\]
(c) The nucleus classification model was evaluated by adopting Accuracy, ROC curve, and AUC values, which are commonly used in the field of image classification.
  Accuracy: the ratio of correctly classified samples to the total number of samples
(6) \[\text{Accuracy}=\frac{{n}_{\text{correct}}}{{n}_{\text{total}}}.]\]
  ROC curve: vertical axis is the true positive rate (TPR) and the horizontal axis is the false positive rate (FPR). It is plotted by sorting the samples according to the prediction results of the classification model, and the samples are predicted as positive examples one by one in this order. Each time the TPR and FPR values are calculated and plotted with their horizontal and vertical coordinates, respectively, to obtain the “ROC curve.” AUC can be obtained by summing the areas of each part under the ROC curve. AUC represents the area under the ROC curve, which takes a floating-point value between 0 and 1.
(d) For clinical validation, we adopted ICC as the evaluation method, i.e., the test consistency of AI diagnosis evaluation results and doctor diagnosis evaluation results.

The ICC was used to evaluate the consistency or reliability of the results of different measures or evaluators for the same quantity.

(7) \[\text{ICC}=\frac{{\sigma }_{\text{T}}^{2}}{({\sigma }_{\text{T}}^{2}+{\sigma }_{\text{B}}^{2}+{\sigma }_{\text{E}}^{2})},]\]

where \[{\sigma }_{\text{T}}^{2}]\] is the variation in the measure, \[{\sigma }_{\text{B}}^{2}]\] is the difference caused by systematic error, and \[{\sigma }_{\text{E}}^{2}]\] is the difference caused by random error. Larger ICC means less variation due to systematic and random errors. The ICC value is between 0 and 1. It is generally accepted that ICC greater than 0.75 is better in terms of consistency, 0.40–0.75 is average and less than 0.40 is worse.

## 3 Experiment results and analysis

### 3.1 Model evaluation

The distribution of the labeled training and validation sets used for each model is shown in Figure 1b. In order to verify the effect of each training model, we validated the trained models on the corresponding labeled validation set. The model training effectiveness was evaluated on the validation set according to the evaluation metrics. The tumor region segmentation model was evaluated based on the mDice coefficient, the tumor region nucleus detection model based on the mAP metric, and the nucleus classification model based on the accuracy metric.

In addition, we compared the results of the individual algorithm of the Ki67 breast quantitative analysis system with those algorithms of SOTA performance in the related field. Specifically, we compared the K-Net+Swin segmentation algorithm that we used with the Segformer [37] algorithm and PSPNet+U-Net algorithm for the tumor region segmentation algorithm, which have advanced performance. For the tumor region nucleus detection algorithm, we compared the SCNet+DCN+LSJ detection algorithm that we used with the HTC algorithm, Cascade_Mask_RCNN algorithm, and Mask_RCNN algorithm, which have advanced performance. For the tumor region nucleus classification algorithm, we compared the PS-ViT classification algorithm that we used with the mainstream ResNet50 algorithm and Vgg16_bn algorithm. Results are shown in Figure 4.

In Figure 4a, we present the results comparison of the tumor region segmentation models. We comprehensively evaluated the average Dice coefficient and the inference speed frame per second. It can be observed that the PSPNet+U-Net algorithm based on a pure convolutional neural network achieved a higher inference speed but with the lowest accuracy of 82.09. In contrast, the Segformer algorithm based on the Transformer achieved a higher average Dice coefficient (83.37) but with a significant decrease in inference speed. The proposed K-Net+Swin segmentation algorithm, combining the windowed Swin Transformer algorithm with a convolutional neural network, maintained a high inference speed, surpassing the pure Transformer-based Segformer, and achieved the best average Dice coefficient of 84.8.

Figure 4b illustrates the results comparison of the tumor region cell detection models using the P–R curve evaluated at IoU 0.5. Cascade_Mask_RCNN and Mask_RCNN algorithms performed similarly and less effectively, indicating that a simple cascade algorithm cannot address the sample consistency issue. To address this problem, the designed HTC algorithm and SCNet+DCN+LSJ algorithm achieved high performance, with SCNet+DCN+LSJ showing the best results.

In Figure 4c, the results comparison of the tumor region cell classification models is presented using ROC curves and AUC for evaluation. It can be observed that the ROC curve of the PS-ViT classification algorithm completely enveloped those of the ResNet50 and VGG16_bn algorithms. This suggests that the PS-ViT algorithm has an absolute advantage in distinguishing positive cells, as reflected in the AUC score, with PS-ViT having an AUC of 0.9666, higher than ResNet50 (0.9574) and VGG16_bn (0.9331).

### 3.2 Model inference

We inferred each model on the test set and performed breast Ki67 patch-level nucleus recognition and made inference visualization. In addition, we also performed full-image inference visualization on WSI images of breast Ki67. The abovementioned visualized inference results are shown in Figure 5.

### 3.3 Results of clinical validation

**Figure 6:**
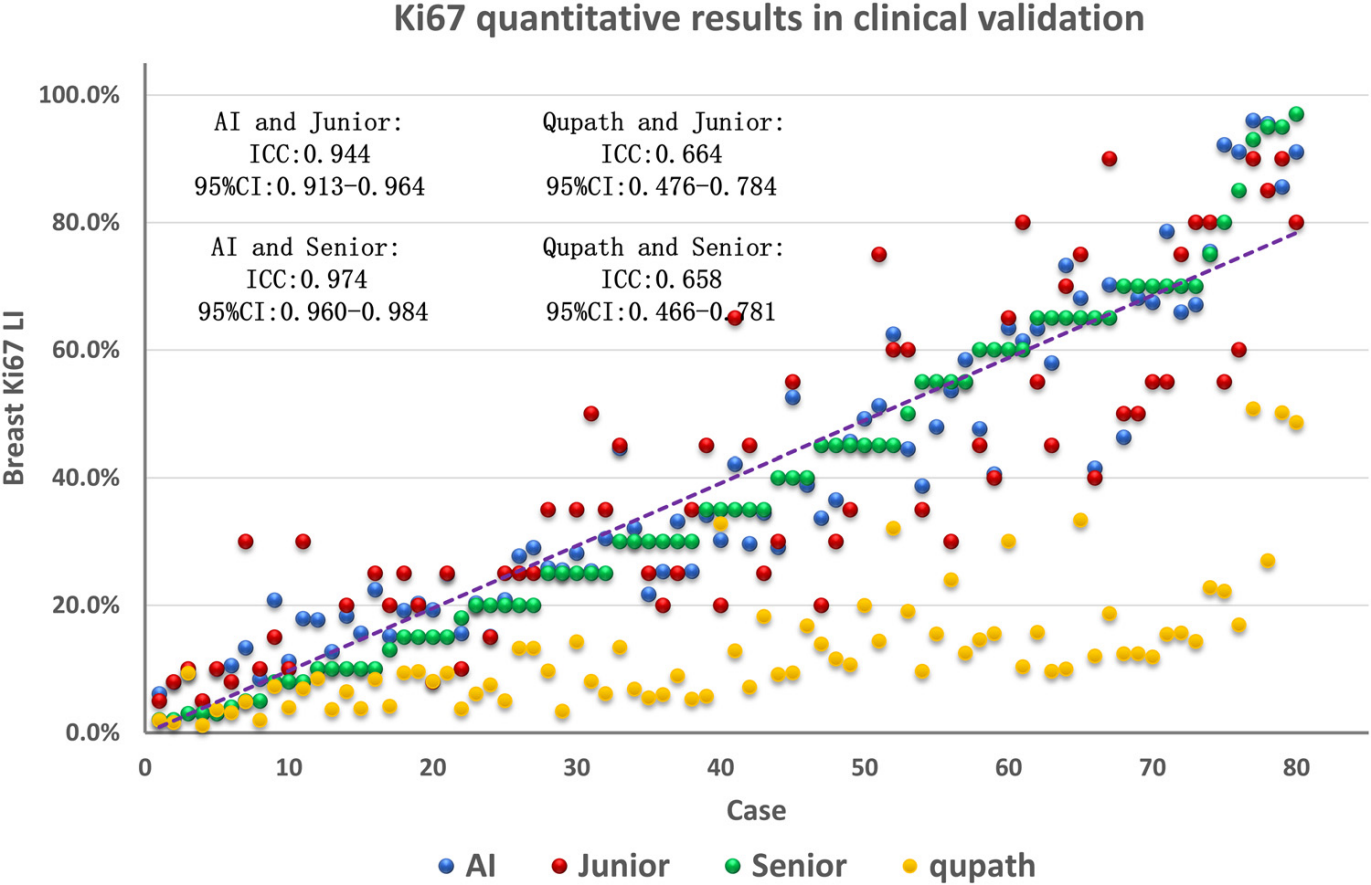
Comparison of clinical validation results and consistency of AI results of the Ki67 quantitative breast analysis system in cases.

We validated our designed breast Ki67 quantitative analysis system comparing the results with the diagnostic results of clinical pathologists. Clinical validation data retrospectively selected a total of 80 patients with invasive breast cancer who underwent Ki-67 IHC examination in Hangzhou Obstetrics and Gynecology Hospital. The selection criteria for these patient data were that modified radical breast cancer specimens and histologically diagnosed patients with IDC. First, the 80 pathology slides were diagnosed by two experts, including a senior pathologist and a junior pathologist, with the junior pathologist having under 5 years of experience in pathology and the senior pathologist having up 10 years of experience in pathology. Then, we input these 80 Ki67 pathology slides into our AI-based breast Ki67 quantitative analysis system to get inference results. Finally, we performed a human and machine comparison of the diagnostic consistency. The results showed that the ICC of diagnosis result between the breast Ki67 quantitative analysis system and pathologists reached 0.964 (95% CI: 0.948–0.976), in which, the ICC of the breast Ki67 quantitative analysis system and senior physician reached 0.974 (95% CI: 0.960–0.984), and the ICC of the breast Ki67 quantitative analysis system and junior physician reached 0.944 (95% CI: 0.913–0.964). The results of the clinical validation are shown in Figure 6. The diagnosis results of our system are mostly in between the results of the senior and junior physicians, and were much closer to those of the senior physicians.

**Figure 7:**
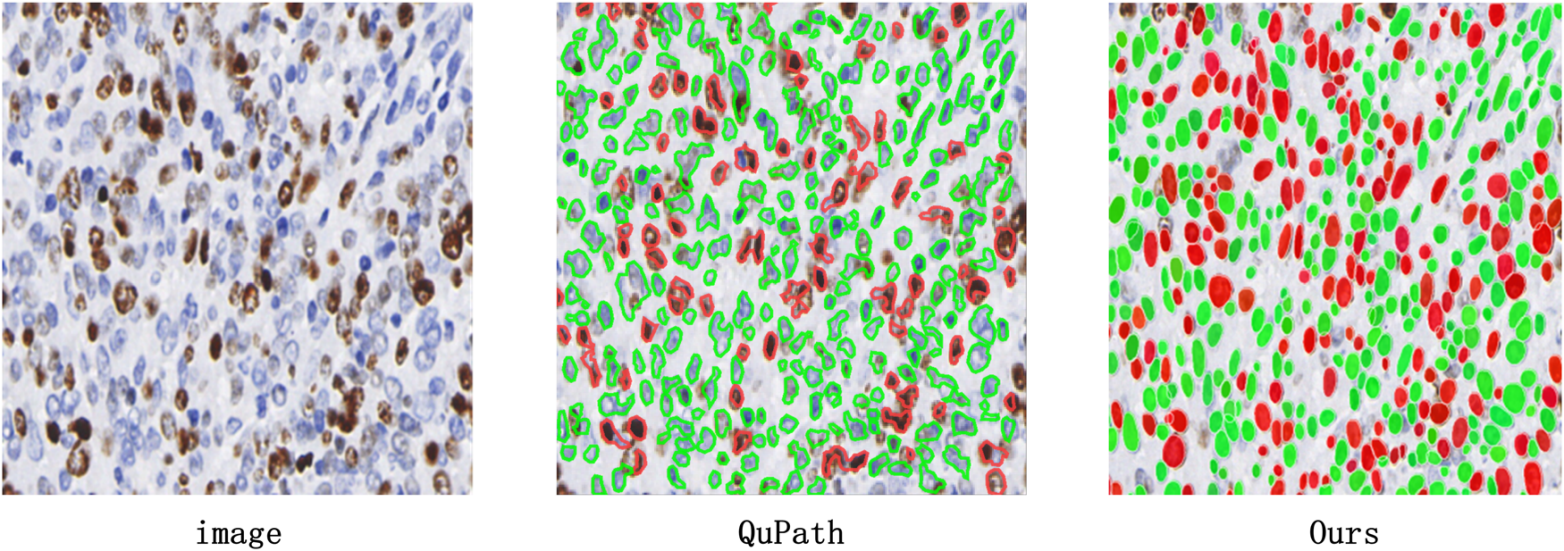
Comparison of QuPath detection results. There are 172 positive cells in the raw image. Only 76 positive cells are detected by QuPath, while our method is able to accurately identify 154 positive cells. Compared to the QuPath, our method performs twice as well. The detection accuracy of our method is 89.5%.

We compared our designed breast Ki67 quantitative analysis system with QuPath v0.4.4 software. QuPath is an innovative bioimage analysis tool for digital pathology and whole-slide image analysis, offering comprehensive tumor identification and high-throughput biomarker assessment tools. Analyzing breast cancer WSI with QuPath software, as shown in Figure 7, we observed that QuPath excelled in accurately detecting normal cells (outlined in green), both in quantity and morphological contour. However, concerning positive cells (outlined in red), QuPath missed a considerable number of visibly positive cells, which were not difficult to distinguish. Additionally, QuPath often failed to accurately outline the edges of identified positive cells. As shown in Figure 6, the diagnostic consistency test results comparing QuPath’s inference results with expert assessments revealed an ICC of 0.658 (95% CI: 0.466–0.781) for diagnostic consistency with senior physicians and an ICC of 0.664 (95% CI: 0.476–0.784) with junior physicians. It is noteworthy that QuPath’s diagnostic results are generally lower than the scores provided by experts, directly related to the presence of numerous false negatives in QuPath software.

**Figure 8:**
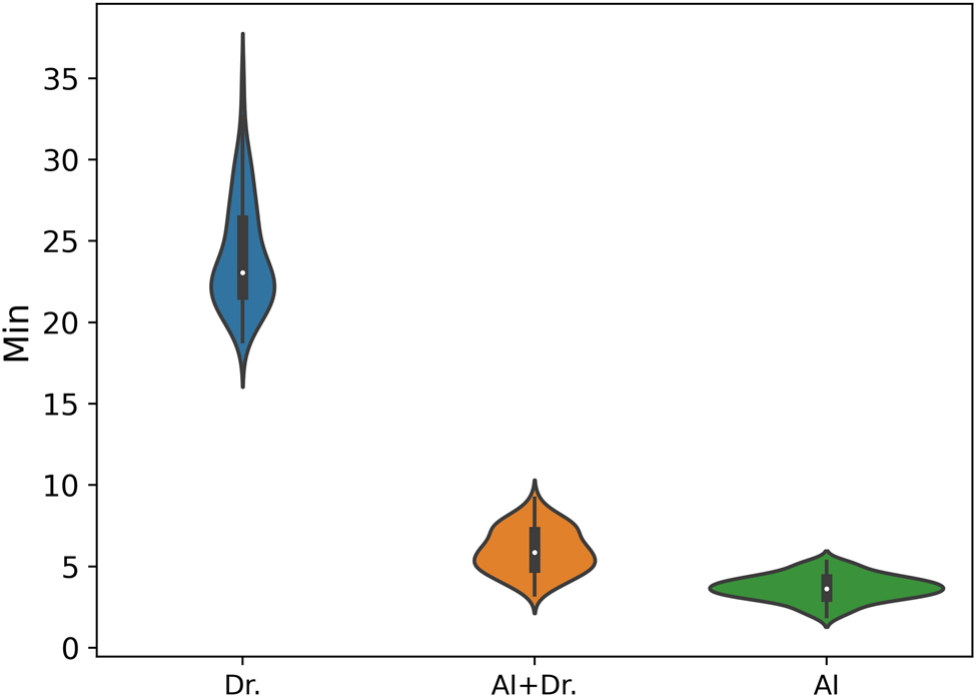
Time efficiency comparison between Doctors, Doctors+AI, and AI. We used three different markers to represent different types of diagnostic times. The horizontal axis represents the three diagnostic ways, i.e., Dr, AI, and AI+Dr. Among them, “Dr.” stands for the time required for a doctor to diagnose alone; “AI+Dr” refers to the time spent by a doctor using our provided analysis system to assist in diagnosis; while “AI” is specifically used to represent the time, it takes for our analysis system to complete the analysis alone.

As an AI-assisted system, the ultimate goal of the breast Ki67 quantitative analysis system is to provide diagnostic assistance to professional doctors rather than replace them in the diagnostic process. Therefore, assessing whether doctors can improve diagnostic efficiency with the assistance of the AI system is a crucial factor in evaluating AI-assisted systems. We conducted a time comparison between the breast Ki67 quantitative analysis system, doctors diagnosing individually, and doctors diagnosing with the assistance of the AI system, and the results are shown in Figure 8. From the figure, it can be observed that doctors typically require approximately 22 min to diagnose a case. When doctors face uncertain situations or need to make conclusions at the threshold boundaries between negative and positive diagnosis, they tend to be more cautious, resulting in significant time and effort consumption. In contrast, AI diagnosis takes only about 3 min, with the inference time mainly influenced by the size of WSI and the number of cells within them. When doctors diagnose with the assistance of the AI system, the diagnostic time is reduced to approximately 5 min. This is because the AI system almost never misses positive cells, and doctors only need to focus on analyzing cells that are challenging to judge based on AI suggestions, ruling out false positives, and providing a comprehensive assessment based on their professional knowledge. This time comparison indicates that AI-assisted systems can significantly improve doctors’ diagnostic efficiency, reduce diagnostic time, allow doctors to address challenging situations in a more target-oriented manner, and maintain control over the overall diagnostic process.

## 4 Conclusion and discussion

In this study, we have developed a complete AI-based breast Ki67 quantitative pipeline that can automatically quantify breast Ki67 nucleus and compare the differences between three Ki67 counting methods of high seniority, low seniority, and full-automatic AI-based through real pathological scenarios. Results showed that the ICC of AI and junior physician was 0.944 (95% CI: 0.913–0.964), and that of AI and senior physician was 0.974 (95% CI: 0.960–0.984). Through the analysis of the related statistical data by Ki67 counting, we found that the Ki67 assessment counts of senior and junior pathologists were quite different. Certainly, there are disparity between the junior pathologists with low seniority, less accumulated number of slide viewings and work experience, and the senior pathologists with high seniority and rich experience in the interpretation of slides with more interference. Therefore, the diagnostic results of senior physicians have higher reference value. It is gratifying that AI-based Ki67 assessment counts were intermediate between senior and junior pathologists and were more relevant to senior pathologists. This illustrates the small difference in gold standard results between AI and manual counts (or AI-based Ki67 count results are between high and low senior pathologists, which also indicates a better consistency between manual and AI methods. The pathology interpretation results are highly consistent while minimizing human-induced interpretation errors, resulting in an accurate and objective ratio of positive Ki67 expression nucleus.

The goal of AI has always been not to replace the work of pathologists, but to improve diagnostic accuracy, reduce human error, and increase efficiency and repeatability. Pathologists can choose the appropriate AI-assisted diagnostic models according to the needs of relevant scenarios. It is important to let AI and pathologists complement each other, improve the consistency and accuracy of breast cancer Ki67 diagnosis among different doctors, provide clinical pathologists with a new teaching model combining digital pathology and AI, improve interpretation ability of pathologists, and provide a reliably scientific and theoretical basis for accurate diagnosis and precise individualized treatment for patients.

While this study presents encouraging results in the prediction of the Ki67 protein biomarker in breast cancer, it also faces some limitations, with a particular focus on generalization ability and the challenges associated with multicenter data. First, the model’s generalization ability is constrained. Deep learning models typically require a substantial amount of training data to achieve good generalization on new data. In the context of quantitative analysis of breast Ki67, acquiring a sufficient amount of annotated data can be a challenging task. We evaluated the breast Ki67 quantitative analysis system using a limited number of cases (80 cases), which may result in some overfitting, leading to potential slight biases in the evaluation results. Second, the challenge of multicenter data is significant. Medical data often originate from different medical centers, with potentially significant differences in data distribution and characteristics. This can make it challenging to reuse a well-trained model across different medical centers. The data used in this study are from a single center, and although we employed data augmentation (such as color transformations) to cover various clinical practices and equipment differences, as well as stain standardization to mitigate distribution gaps in multicenter data domains, careful consideration and reevaluation are still necessary for data from other centers, especially WSI with significant staining variations, to plan the next steps. Additionally, the quality of WSI data itself is a potential limitation.

Currently, AI-assisted diagnosis has gradually been widely used in the field of pathology, but there are still many issues that need to be addressed in order to make it clinically applicable and truly widespread. First, pathology techniques such as HE slide preparation and IHC staining are experiment schemes determined by multiple steps and factors. The normalization, standardization, and strict quality control of pathology slides are the prerequisites for the promotion of the application of computer-aided diagnosis technology. Second, only by solving the problem of diagnosis standardization and unifying the counting habits and methods of pathology diagnosticians in clinical pathological practice, can we reduce the differences caused by individualization in manual counting and provide an appropriate reference way for the development of AI-assisted diagnostic tools. Only by gradually solving these problems can AI-assisted diagnosis be better used by pathologists.

In conclusion, the research results show that our breast Ki67 quantitative analysis system has achieved excellent results and may lead to highly repeatable and platform-independent scores in Ki67 evaluation. We believe that this study features an important step in the systematic, intelligent application of AI to Ki67 and it is a key step in the application of AI to breast Ki67 in clinical care. In the future, a multi-institutional study is underway to further demonstrate clinical efficacy and utility.

## Supplementary Material

Supplementary material
