# Supplementary material for: Quantitative immunohistochemistry analysis of breast Ki67 based on artificial intelligence
Source: Open Life Sci. 2024 Dec 31;19(1):20221013. doi: 10.1515/biol-2022-1013 (PMC11751672; doi:10.1515/biol-2022-1013)
Supplement: Supplementary material [file biol-2022-1013-sm.pdf]

Supplementary material

S1 Data collection and annotation

The data source for this study was the Hangzhou Obstetrics and Gynaecology Hospital. A total of 400 breast Ki67 WSI pathology slides were obtained scanning by KFBIO Scanner. The WSI pathology slides were divided into 8:2, with 320 slides used for model data annotation and 80 slides used for model inference. The data division is based on patients, meaning that the inference dataset used for testing is entirely independent of the annotation dataset. There are no common patients, and any WSI region comes from the annotation dataset used for training. The annotation dataset includes masks indicating the regions of positive cells and their Ki67 percentage annotations. In contrast, the dataset used for inference only contains Ki67 percentage annotations.For the WSI to be annotated, pre-processed slicing was used to cut the slide into 1,536 × 1,536 pixel images. Then the dataset for the tumor region segmentation model, tumor region nucleus detection model, and tumor region nucleus classification model were constructed respectively. The dataset was divided in the ratio 7:2:1 (training:validation:test), while the training set was used for model training, the validation set was used to evaluate the effect of the model, and the test set was used for model inference. Specifically, the division of the annotated dataset is mixed based on tiles/patches. This implies that the training set, validation set, and test set in this portion may come from the same patient, but they cannot originate from the same WSI region. The data division process is illustrated in Figure S1.

S2 Tumor region segmentation

Tumor region segmentation is a fundamental step of the quantitative breast Ki67 analysis system, which can segment the tumor regions, eliminating non-tumor regions and blank regions, thereby improve the rate of tumor cell identification.

We used a semantic segmentation method combining Swin and K-net[16] for tumor region segmentation, considering the K-net network achieved state of the art performance in the field of image segmentation. Meanwhile, due to the characteristic of the semantic segmentation task, we can keep the number of kernels consistent with the number of semantic class and make each kernel responsible for the generation of a fixed semantic class mask.

The semantic segmentation task basically assigns each pixel to a predefined group. As the groups in an image are

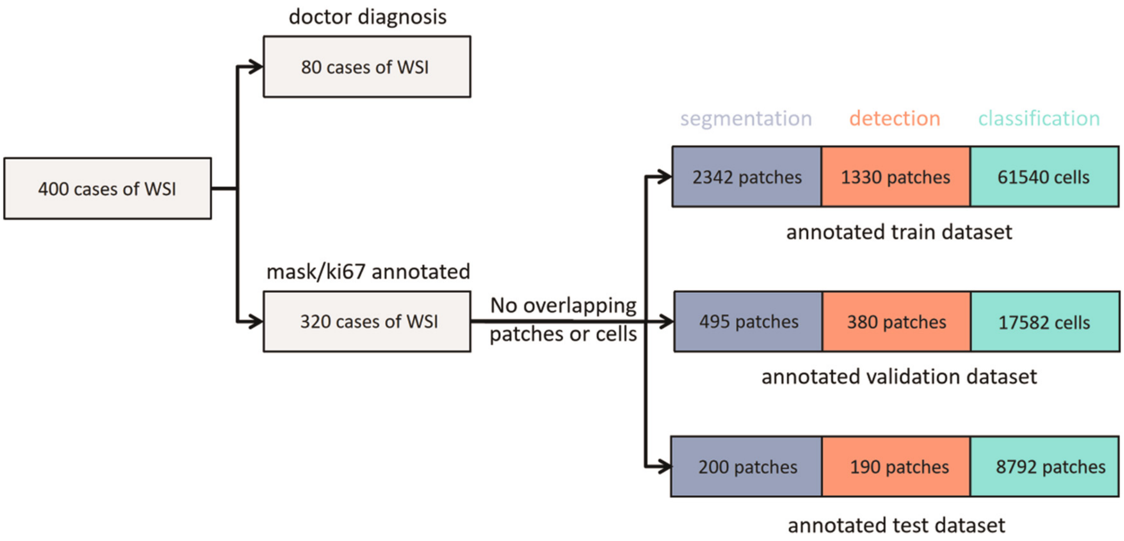

Figure S1: Data segmentation process.

usually finite, the maximum number of groups for the segmentation task can be set to N. In this way, semantic segmentation has N predefined semantic classes. Therefore, the image can be divided into N groups using N kernels and each kernel is responsible for finding the pixels belonging to its corresponding group.

Specifically, given a neural network output feature map $F \in R^{B\times C\times H\times W}$, in which B, C, H and W are the number of images, the number of channels, height, and width of the feature map respectively. Only N kernels $K \in R^{N\times C}$ are needed to convolve it to obtain the corresponding segmentation prediction $M \in R^{B\times N\times H\times W}$ and hence obtain the segmentation result.

$$M = \sigma(K \times F), \tag{1}$$

where σ is the softmax function to assign each pixel to only one of the kernels.

K-Net can adapts to different frameworks by making the semantic convolution kernel dynamic. For example, K-Net significantly improves the FCN (6.6 mIoU). The combination of Swin Transformer and K-Net outperforms PSPNet and UperNet by 0.7 and 0.9 mIoU respectively, and achieves performance comparable to DeepLabv3. Furthermore, the effectiveness of K-Net does not saturate under very strong model representation, and it still brings significant improvements even though compared to UperNet and Swin Transformer.

S3 Nucleus detection in tumor region

SCNet proposes effective sample consistency by using a single deep mask branch instead of multiple shallow mask branches, solving the previous problem of all mask branches in the network being isolated from each other without direct connections, and the problem of noise frames mask prediction on intermediate process. The effective sample consistency proposed by SCNet significantly improves the network speed on top of the improved accuracy. In addition, the SCNet network takes into account feature relay and global contextual information, using implicit interaction information between MBB and mask branches to improve overall performance. Meanwhile, the relay feature explicitly combines the MBB feature with the mask feature, which in turn improves the prediction of the mask. Relay feature exploits the relationship between detection and segmentation subtasks to make the box feature provide prior information for mask branches, while mask prediction in turn supervises and refines box feature through backpropagation. This tight coupling can greatly help improve our performance. For the global contextual information, SCNet takes backbone features as input and outputs multi-label predictions and global contextual features. First, the top features of the FPN are fed into two consecutive blocks of residuals, followed by global averaging pooling. Pooling layer features are used for two sub-branches, namely multi-label classification and global contextual features. Multi-label prediction is supervised by all known objects in the image, and contextual features are used to fuse with box and mask features.

S4 Nucleus classification in tumor region

PS-ViT proposes a progressive sampling (PS) module to address the problems posed by the direct division of patches. The PS module samples the image in an iterative way, which is used directly in ViT network based on the transformer. The PS module takes point sampling from the input and predicts an offset for every sample point at each iteration. The offset updates the sample location so that each sample point can progressively approximate a key region in the image.

S5 Training implementation

The algorithm framework involved in the Ki67 breast quantitative analysis system is achieved based on Python 3.7 and Pytorch 1.8.0. For all training samples, data augmentations such as flips and rotations are used to increase data diversity. We train our models on a NVIDIA RTX 3090 *4 GPU with 24GB memory. The input image size of the tumor region segmentation and tumor region nucleus detection are set as 512 × 512, while the input image size of tumor region nucleus classification is 48 × 48. During the training period, it should be pointed out that image enhancement methods such as image LSJ large scale dithering is used for tumor region segmentation. We train it with 80,000 iterations. The batch size is 4 and the better AdamW optimizer with weight decay 5×10^{-4} is used to optimize the tumor region segmentation model. The initial learning rate is 0.0005, and it is decayed by 0.1 after 60,000 and 72,000 iterations, respectively. For the tumor region nucleus detection, the popular SGD optimizer with momentum 0.9 and weight decay 1×10^{-4} is used to optimize the tumor region nucleus detection model. The model is trained with a batch size of 4 for 50 epochs. The learning rate is 0.03, and it is decreased by 0.1 after 36 and 48 epoch respectively. For the tumor region nucleus classification, the SGD optimizer with

Table S1: Comparison of the system proposed in this article with other research methods

Method	Basic architecture	Number of stage	Instance segmentation	Expressive ability	Complexity level
IHC-Net	CNN	One	No	Weak	Simple
Mask R-CNN	CNN	Two	Yes	Weak	Moderate
DDTNet	CNN	One	No	Weak	Moderate
Ours	Attention+CNN	Three	Yes	Strong	Complex

momentum 0.9 and weight decay 5e-4 is used to optimize the tumor region nucleus classification model. The model is trained with a batch size of 32 for 70 epochs and the learning rate is 0.00001.

S6 Cell/tissue segmentation for breast cancer

Currently, there are studies that have applied deep learning-based methods to the segmentation of breast cells or tissues. This section will compare the proposed method in this paper with other methods. Mahanta, et al. proposed a deep learning-based nuclear segmentation and comprehensive classification scheme called IHC-Net for quantitatively assessing hormonal states on IHC specimens [1]. This method adopts the basic architecture of an encoder-decoder, similar to UNet, and optimizes aspects such as network depth and upsampling. Amgad et al. used the Mask R-CNN framework for instance segmentation at the tissue and cell levels [2]. Zhang et al. introduced a dense dual-task network named DDTNet, capable of automatic detection and segmentation in histopathological images [3]. We compare these methods with the system proposed in this paper, highlighting some key designs in Table S1.

S7 Qupath parameter settings

Table S2: In the experimental procedure of this article, the parameter configuration information of the QuPath software called is recorded and set in detail

Parameters		Value
Setup parameters	Detection image	Hematoxylin OD
	Requested pixel size	0.5 μm
Nucleus parameters	Background radius	8 μm
	Median filter radius	0 μm
	Sigma	1.5 μm
	Minimum area	10 μm^2
	Maximum area	400 μm^2
Intensity parameters	Threshold	0.1
	Max background intensity	2
	Split by shape	Yes
Cell parameters	Cell expansion	5 μm
	include cell nucleus	Yes
General parameters	Smooth boundaries	Yes
	Make measurements	Yes

References

[1] Mahanta LB, Hussain E, Das N, Kakoti L, Chowdhury M. IHC-Net: A fully convolutional neural network for automated nuclear segmentation and ensemble classification for Allred scoring in breast pathology. Appl Soft Comput. 2021;103:107136.
[2] Amgad M, Atteya LA, Hussein H, Mohammed KH, Hafiz E, Elsebaie M, et al. NuCLS: A scalable crowdsourcing approach and dataset for nucleus classification and segmentation in breast cancer. GigaScience. 2022;11:giac037.
[3] Zhang X, Zhu X, Tang K, Zhao Y, Lu Z, Feng Q. DDTNet: A dense dual-task network for tumor-infiltrating lymphocyte detection and segmentation in histopathological images of breast cancer. Med Image Anal. 2022;78:102415.
